# Occupational risk of COVID-19 related hospital admission in Skåne, Sweden: A register-based cohort study

**DOI:** 10.1371/journal.pone.0335662

**Published:** 2025-11-04

**Authors:** Jesse D. Thacher, Andreas Vilhelmsson, Sandra S. Tøttenborg, Jens Peter Ellekilde Bonde, Kajsa U. Petersen, Esben M. Flachs, Christel Nielsen, Kristina Jakobsson, Kerstin Nilsson, Luise M. Begtrup, Hannah N. Frankel, Lars Rylander

**Affiliations:** 1 Division of Occupational and Environmental Medicine, Department of Laboratory Medicine, Lund University, Lund, Sweden; 2 Department of Occupational and Environmental Medicine, Copenhagen University Hospital - Bispebjerg and Frederiksberg, Copenhagen, Denmark; 3 Department of Public Health, Section of Environmental Health, University of Copenhagen, Copenhagen, Denmark; 4 Department of Public Health, University of Copenhagen, Copenhagen, Denmark; 5 Department of Public Health and Community Medicine, Sahlgrenska Academy, University of Gothenburg, Gothenburg, Sweden; 6 Department of Public Health, Kristianstad University, Kristianstad, Sweden; Ruhr University Bochum, GERMANY

## Abstract

**Purpose:**

Given the paucity of data regarding workplace risk of COVID-19, particularly from countries with limited lockdowns, we aimed to quantify the occupational risks of COVID-19-related hospital admission among workers in Sweden.

**Methods:**

We identified 607,179 employed individuals, 20−69 years of age, in Skåne, Sweden. From December 31^st^, 2019—December 31^st^ 2021, 2,633 incident COVID-19-related admissions were identified. Using a job exposure matrix for risk of becoming infected with the SARS-CoV-2 virus in an occupational setting we delineated occupations with low work-related risk. Based on these reference occupations, incidence rate ratios (IRR) and 95% confidence intervals (CI) were computed by Poisson regression for four-digit occupations defined by the International Standard Classification of Occupations job codes (ISCO-08).

**Results:**

After adjusting for various sociodemographic characteristics, risk compared to reference occupations was elevated among healthcare occupations as a group (IRR 1.31; 95% CI: 1.13–1.51), with nurses, healthcare assistants, and nursing aids having the highest IRRs (ranging from 1.28–1.54). In the educational sector, no apparent elevated overall risk was observed (IRR 1.03; 95% CI: 0.86–1.23). For the transportation sector, an overall excess risk was observed (IRR 1.34; 95% CI: 1.10–1.65), with bus and tram drivers having the highest risks. IRRs < 1 were observed among electricians, some builders, and software developers.

**Conclusion:**

Excess risk of COVID-19-related hospital admission was observed in many patient-facing occupations across the healthcare sector and in multiple occupations within the transportation sector. However, despite limited lockdowns and legislation, no apparent increased risks were observed in the educational or retail sales sectors.

## Introduction

During the COVID-19 pandemic, workplaces were important contributors to the spread of severe acute respiratory syndrome corona virus 2 (SARS-CoV-2 virus) [1,2]. As with most communicable diseases, SARS-CoV-2 may constitute an occupational risk to workers [3–9]. Occupational risk of developing COVID-19 not only depends on personal risk factors and vulnerability, but likely varies substantially across occupations and environments. While some studies have explored the occupational risks of COVID-19 [6,10,11], we lack clear insight into which occupations have the highest risks of contracting COVID-19 in countries with limited legislation and no sweeping mandatory lockdowns.

There is likely considerable variation between the patterns across countries depending on the extent of lockdowns, sector specific regulations, as well as guidance and the risks in the context of less mandatory lockdowns remain to be elucidated. Sweden stands out from other nations with its comparatively limited regulations on social distancing and mask-wearing, as well as its decision to keep restaurants, primary schools, and daycare centers open [12], particularly in contrast to its Nordic neighbors, which imposed more strict lockdown measures.

Enhancing the understanding of occupational risks under fewer lockdowns and restrictions would significantly assist authorities in determining whether specific activities within certain sectors should be restricted in efforts to mitigate the transmission of SARS-CoV-2 and avoid shutting down occupations with little excess risk. Furthermore, it is essential for directing public health efforts towards curtailing future epidemics transmission of airborne viruses and enhancing safeguards for workers’ wellbeing.

To this end, we aimed to quantify the occupational risks of COVID-19-related hospital admission among workers in a Swedish population with limited legislation and lockdowns during the first two years of the pandemic.

## Methods

### Study population

The study population was identified from the Swedish Total Population Register (RTB) and the Occupational Register (Statistics Sweden) and comprised all employed individuals ages 20–69 years residing in Skåne County on December 31^st^, 2019. Skåne county is the southernmost county in Sweden.

### Occupation assessment

Occupational codes were extracted from the Swedish national socioeconomic database – LISA (the longitudinal integration database for health insurance and labor market studies, compiled by Statistics Sweden, accessed 08-12-2022) [13]. The occupation held in November 2019 was classified by the Swedish version of the International Standard Classification of Occupations (SSYK-2012) and includes a four-digit job code [14]. No data on changes in occupation from 2020 to 2021 was available in the present study. We converted Swedish codes to the international classifications ISCO-08 codes. Apart from a handful of job codes, the Swedish classifications are nearly identical to the international classifications. For example, the Swedish code for graphic designers is 2171 whereas in ISCO-08 it is 2166.

### Outcome

Incident cases of COVID-19 related hospital admissions between December 31^st^, 2019, and December 31^st^, 2021, were identified using the Skåne Health Care Database (Region Skånes Vårddatabas, RSVD, accessed 01-18-2023) [15]. Since the early 2000s, the RSVD has encompassed the entire population of Skåne county and serves as a resource for descriptive, etiological, and health economic research. The RSVD covers all levels of care delivered in the county and has nearly 100% coverage and good validity [15]. Up to 15 diagnoses for each admission can be registered, and the first registered diagnosis describes the principal reason for the admission. COVID-related hospital admissions were defined according to the *International Classification of Diseases (ICD)* version 10 as: U07.1 (COVID-19, virus identified) or U07.2 (COVID-19, virus not identified, clinically diagnosed) in any diagnostic position. On December 27^th^, 2020, the first vaccinations against COVID-19 began. It should be noted that in Sweden healthcare is universally accessible to all residents.

### Reference group

We defined the reference group *a priori* according to an expert developed COVID-19 Job Exposure Matrix (JEM) [16] and included all occupations with unlikely occupational exposure to SARS-CoV-2, as defined by the JEM score of zero (S1 Table). The independent expert-rated COVID-19 JEM has been described in detail earlier, but was designed to capture eight dimensions which were considered key risk factors of contracting the virus (e.g., working from home or not working with others) [16]. We defined the reference based on a sum score of zero across the eight determinants of occupational SARS-CoV-2 exposure (with a possible range of zero to 24). The reference group comprised 40 occupations (defined by four-digit ISCO-08 codes) and the three most frequent occupations in the reference group were production clerks, commercial sales representatives, and statistical and mathematical related professionals (33.2% of the reference group, ISCO-08 codes 4322, 3322, and 3314, respectively).

### Covariates

Data on demographic and social characteristics were obtained from the RTB and LISA registers and included age, sex, country of birth, and number of household members. Dates of COVID-19 vaccinations were obtained from the Public Health Agency of Sweden.

### Statistical analysis

We used Poisson regression models applied to counts of incident (first) COVID-19 hospital admissions to estimate incidence rate ratios (IRR) and corresponding 95% confidence intervals (CI) in relation to occupation for all non-referent occupations with >500 employees. Follow-up began on December 31^st^, 2019, and subjects were censored at the date of COVID-19 admission, death, retirement, or December 31^st^ 2021, whichever came first. We considered markedly increased effect estimates to be relevant, even if they were not statistically significant.

Crude and adjusted IRRs were calculated for COVID-19-related admissions in association with occupation in two models: Model 1 adjusted for age (10 year categories) and sex, and Model 2 with further adjustment for education (short – primary or lower secondary education, medium – upper secondary or post-secondary education less than two years, long – post-secondary education two or more years or postgraduate education), country of birth (Sweden, other western countries, eastern Europe, other), number of household members (0, 1, 2, 3, 4+), and COVID-19 vaccination (as a time varying covariate, from date of second vaccination until end of follow-up). Occupations with ≤500 employees and participants with missing ISCO-08 codes were assessed as separate categories. Models 1 and 2 were performed as complete case analyses with the same population in all analyses.

Lastly, we conducted sensitivity analyses based on Model 2 including only cases with a COVID-19 diagnosis in diagnostic position one, representing the principal reason for hospital admission and likely more severe COVID-19. Similarly, we performed analyses using only lab-confirmed COVID-19 diagnoses (diagnostic code U07.1) as the case definition.

Statistical analyses were performed in SAS 9.4 (SAS Institute Inc., Cary, NC, USA).

The following study was approved by the Swedish Ethical Review Authority (Dnr: 2022-01359-01) and conformed with the Helsinki Declaration. Data was fully anonymized prior to analysis.

## Results

**Fig 1:**
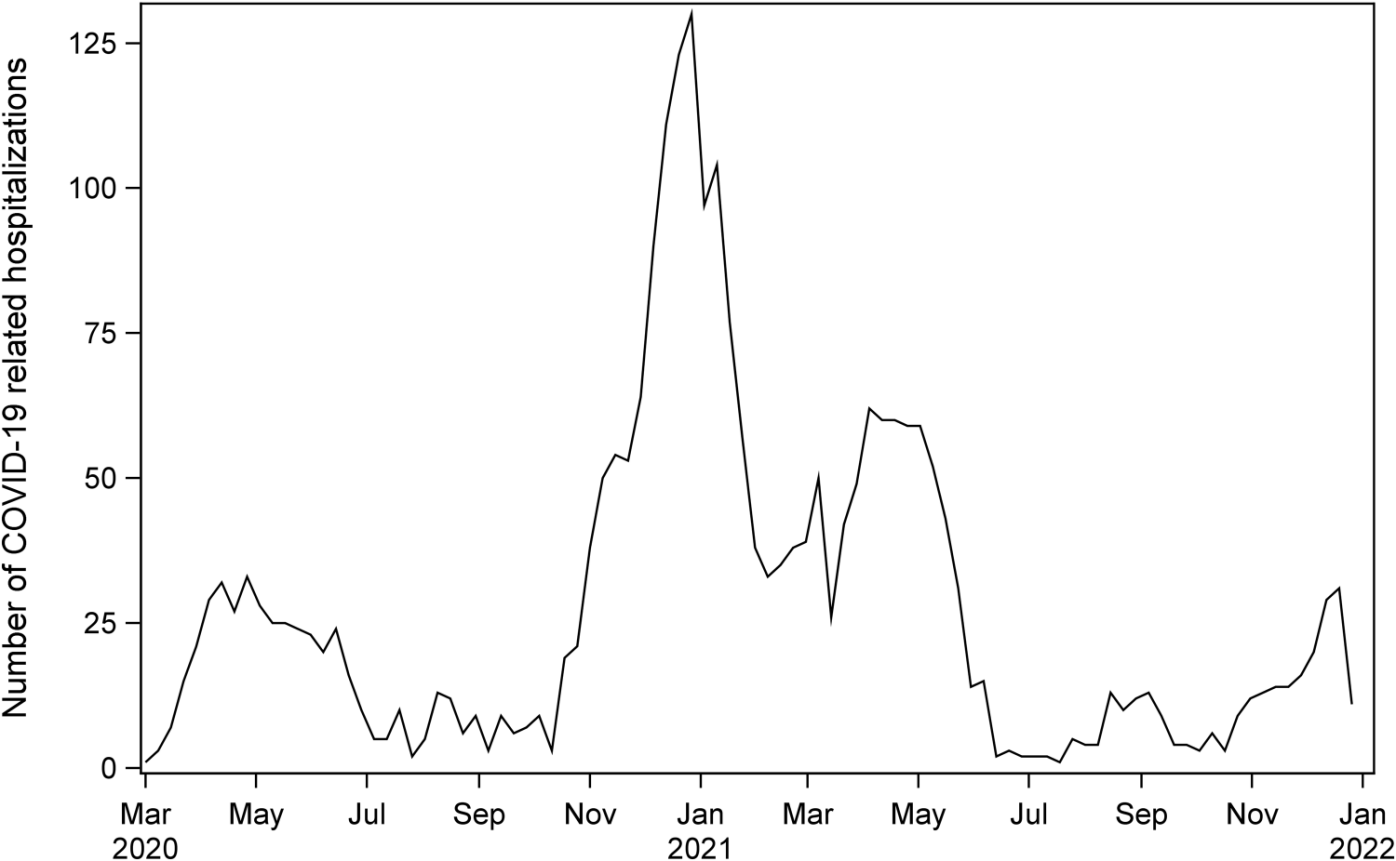
COVID-19 related hospital admissions among employees ages 20-69 in Skåne, Sweden in 2020 and 2021.

An overview of the number of employees, number of different occupations, and incidence COVID-19-related hospital admissions in the study population is presented in Table 1. Analyses were based on 607,179 individuals, 49% female, encompassing 326 different occupations. The median age was 43 years and 85% were from western Europe. Across the follow-up period, we observed 2,633 incident COVID-19 admissions, which peaked around week 52 of 2020 (Fig 1).

**Table 1:** Overview of the study population (N = 607,179).

	Employees (n)	4-digit ISCO-08 occupations (n)	Incident COVID-19 related hospital admissions (n)
At-risk^a^ occupations with >500 employees	418,681	164	1,820
At-risk^a^ occupations with ≤500 employees	26,756	121	112
Reference occupations^b^	102,168	40	395
Missing occupational codes	59,574	--	306
Entire study population	607,179	326	2,633
Sex			
Males	310,506	326	1,603
Females	296,673	325	1,030

^a^Non-referent occupations.

^b^Low likelihood of occupational SARS-CoV-2 exposure according to an expert rated COVID-19 job exposure matrix with eight dimensions (sum score = 0). Occupations that met this criterion were included in the reference group whatever their number of employees.

The distribution of sociodemographic characteristics among the reference group (JEM sum score = 0) and at-risk occupations (JEM sum score = 1–24) is presented in S2 Table. Among at-risk occupations, it was more common to be male, younger, have short or medium education, and born outside Sweden compared to the reference group. Those with missing occupational codes were more likely to be male, younger, have low education, born outside Sweden, and live alone compared to both the reference and at-risk occupations (S2 Table). Overall, individuals with missing occupational codes are typically associated with firms that have not reported to the authorities, certain types of union work, as well as project-based employment (Ludvigsson et al. 2019).

The associations between COVID-19-related admission and individual-level risk factors are presented in Table 2. As anticipated, following adjustment, risk was generally higher among males, older age groups, shorter education, foreign born, and those with higher household occupancy. Completed vaccination (minimum of two doses) against COVID-19 was associated with lower risk.

**Table 2:** Associations between sociodemographic characteristics and COVID-19 related hospital admission 2020−21.

Characteristic	Employees (n)	Number of COVID-19 hospital admissions	IRR (95% CI)
Sex^a^			
Males	310,506	1,603	1.49 (1.38-1.62)
Females	296,673	1,030	Reference
Age (years)^b^			
20 - <30	114,075	220	Reference
30 - <40	141,433	379	1.39 (1.18-1.64)
40 - <50	143,453	594	2.15 (1.85-2.51)
50 - <60	136,730	908	3.47 (2.99-4.02)
≥60	71,488	532	3.88 (3.31-4.54)
Education^c^			
Low	55,985	368	1.61 (1.42-1.83)
Medium	312142	1,430	1.27 (1.16-1.38)
High	239052	813	Reference
Country of birth^c^			
Sweden	471,509	1,518	Reference
Other western countries	43,806	212	1.52 (1.31-1.75)
Eastern Europe	34,372	366	3.54 (3.15-3.96)
Other	57,492	537	3.49 (3.16-3.85)
Number of household members^c^			
1	176,332	670	Reference
2	165,845	775	1.17 (1.05-1.30)
3	107,427	514	1.48 (1.32-1.66)
≥4	157,575	674	1.43 (1.28-1.60)
Completed vaccination against Covid-19^c,d^			
Yes	333,510	2007	0.47 (0.43-0.52)
No	273,669	626	Reference

^a^Adjusted for age.

^b^Adjusted for sex.

^c^Adjusted for age and sex.

^d^At least two vaccinations.

**Fig 2:**
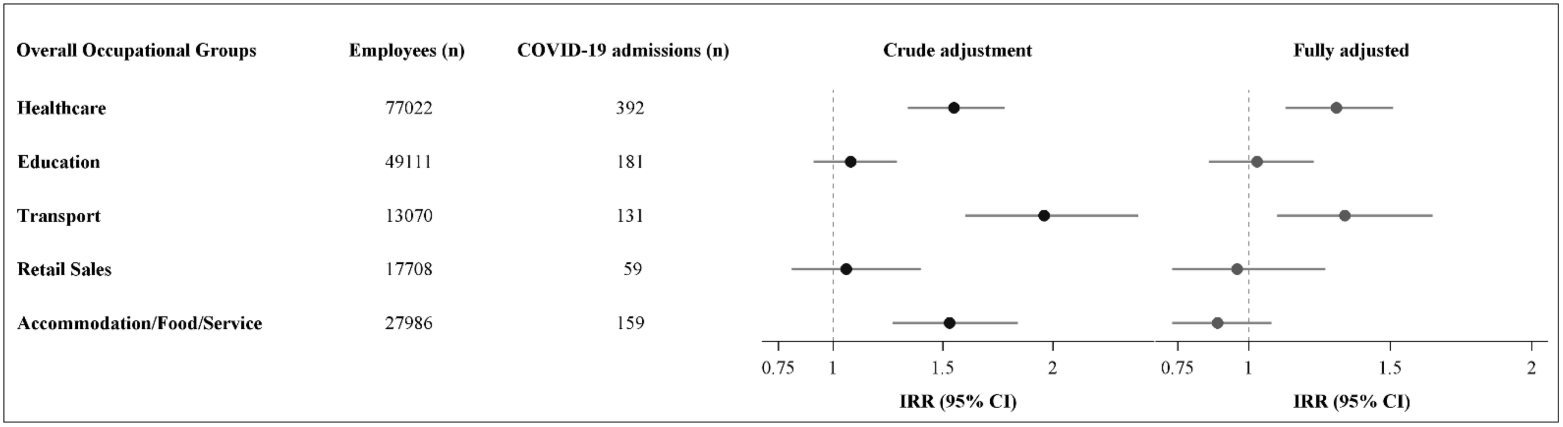
Risk of COVID-19 related hospital admission in relation to occupational group. Crude model adjusted for sex and age (10-year groups). Fully adjusted model adjusted for sex, age (10-year groups), education (3 groups), country of origin (4 categories), number of household members (0, 1, 2, 3, 4+), and COVID-19 vaccination (from date of second vaccination until end of follow-up).

Overall, healthcare occupations as a group had an elevated risk of COVID-19-related admission, with an IRR of 1.31 (95% CI: 1.13–1.51) compared to the reference group (Fig 2).

Within healthcare, three out of 13 occupations with >500 employees had an elevated risk of COVID-19-related admission; nursing professionals (IRR 1.54; 95% CI: 1.16–2.03), healthcare assistants (IRR 1.28; 95% CI: 1.08–1.52), and nursing aides in private homes (IRR 1.42; 95% CI: 1.07–1.88) (Table 3). Some indication of excess risk was also noted for physiotherapists (IRR 1.67; 95% CI: 0.83–3.37), recreational therapists (IRR 1.47; 95% CI: 0.65–3.30), dental assistants and therapists (IRR 1.45 (95% CI: 0.77–2.73), and x-ray technicians (IRR 1.79; 95% CI: 0.74–4.34). Psychological therapists, dentists, and midwifery professionals did not appear to have an elevated risk of COVID-19-related admission.

**Table 3:** Risk of COVID-19 related hospital admission in relation to occupation. Incidence rate ratios (IRR) with 95% confidence limits (CI) relative to employees in all occupations with unlikely occupational exposure to SARS-CoV-2^a^.

Occupation	ISCO-08 code	Employees (n)	COVID-19 admissions (n)	Crude adjustment IRR (95% CI)^b^	Fully adjusted IRR (95% CI)^c^
HEALTHCARE					
Healthcare overall	^d^	77,022	392	1.55 (1.34-1.78)	1.31 (1.13-1.51)
Nursing Professionals	2221	13,225	59	1.35 (1.02-1.78)	1.54 (1.16-2.03)
Medical Practitioners	2211, 2212, 2213, 2219	6,236	29	1.16 (0.79-1.69)	1.13 (0.77-1.65)
Healthcare Assistants	5321	42,596	224	1.67 (1.41-1.97)	1.28 (1.08-1.52)
Physiotherapists	2264	1,717	8	1.39 (0.69-2.79)	1.67 (0.83-3.37)
Medical Laboratory Technicians	3212	1,528	7	1.39 (0.66-2.95)	1.25 (0.59-2.64)
Psychological Therapists	2634	1,450	<5	0.19 (0.03-1.34)	0.22 (0.03-1.54)
Recreational Therapists	2269	1,452	6	1.29 (0.58-2.90)	1.47 (0.65-3.30)
Dentist	2261	835	<5	0.63 (0.16-2.53)	0.55 (0.14-2.23)
Dental Assistants and Therapists	3251	1,880	10	1.67 (0.89-3.13)	1.45 (0.77-2.73)
X-ray Technicians	3211	685	5	1.81 (0.74-4.37)	1.79 (0.74-4.34)
Midwifery Professionals	2222	765	<5	0.77 (0.19-3.10)	0.88 (0.22-3.54)
Nursing Aides (private homes)	5322	8281	59	2.10 (1.60-2.76)	1.42 (1.07-1.88)
Other Hospital related activities	2635	2,608	9	0.97 (0.50-1.89)	0.95 (0.49-1.84)
EDUCATION					
Overall Education	^e^	49,111	181	1.08 (0.91-1.29)	1.03 (0.86-1.23)
Childcare Workers	5311	11,678	52	1.62 (1.21-2.17)	1.11 (0.83-1.49)
Preschool Teachers	2342	11,420	37	1.03 (0.73-1.45)	1.11 (0.79-1.56)
Primary School Teachers	2341	15,163	56	1.07 (0.81-1.42)	1.03 (0.78-1.37)
Secondary Education Teachers	2330	4,146	21	1.22 (0.79-1.89)	1.31 (0.85-2.04)
University and Higher Education Teachers	2310	5,438	9	0.41 (0.21-0.78)	0.45 (0.23-0.88)
Vocational Education Teachers	2320	1,266	6	0.96 (0.43-2.14)	1.03 (0.46-2.31)
TRANSPORT					
Overall Transport	^f^	13,070	131	1.96 (1.60-2.39)	1.34 (1.10-1.65)
Heavy Truck and Lorry Drivers	8332	7,158	45	1.26 (0.92-1.72)	1.12 (0.82-1.53)
Bus and Tram Drivers	8331	3,069	59	3.34 (2.53-4.39)	1.68 (1.27-2.23)
Car, Taxi and Van Drivers	8321	2,249	24	2.12 (1.40-3.21)	1.24 (0.82-1.88)
Locomotive Engine Drivers	8311	594	<5	1.02 (0.33-3.17)	1.11 (0.36-3.47)
RETAIL SALES					
Overall Retail Sales	^g^	17,708	59	1.06 (0.81-1.40)	0.96 (0.73-1.27)
Shop Sales Assistants	5223	13,418	45	1.16 (0.85-1.58)	1.10 (0.80-1.50)
Cashiers and Ticket Clerks	5230	1,547	<5	0.25 (0.03-1.76)	0.20 (0.03-1.39)
Retail Trade Managers	1420	1,402	7	1.02 (0.48-2.14)	0.94 (0.44-1.98)
Pharmaceutical Technicians and Assistants	3213	509	0	--	--
Butchers and Fishmongers	7511	832	6	1.48 (0.66-3.31)	1.01 (0.45-2.27)
ACCOMODATION, FOOD, BUILDING, PERSONAL AND PROTECTIVE SERVICES, RECREATION ACTIVITY					
Overall Accommodation, Food, Building, Personal and Protective Services, Recreation Activity	^h^	27,986	159	1.53 (1.27-1.84)	0.89 (0.73-1.08)
Cooks	5120	5,087	24	1.31 (0.87-1.98)	0.83 (0.54-1.29)
Waiters and Bartenders	5131, 5132	3,135	10	1.34 (0.71-2.50)	1.14 (0.61-2.14)
Protective Service Workers	5411-5419	4,465	19	1.13 (0.71-1.80)	1.03 (0.65-1.63)
Kitchen Helpers	9412	6,967	39	1.93 (1.39-2.68)	1.07 (0.76-1.50)
Cleaners and Helpers	9111	10,749	65	1.74 (1.34-2.26)	0.85 (0.64-1.11)
Building Caretakers	5152	5,183	31	1.11 (0.77-1.61)	0.88 (0.61-1.28)
Hairdressers and Cosmetologists	5141,5142	2,990	12	1.33 (0.75-2.36)	0.91 (0.51-1.63)
Fast Food Preparers	9411	544	9	4.32 (2.23-8.37)	1.76 (0.90-3.42)
Gardeners and Horticultural Growers	6113	2,644	15	1.18 (0.71-1.98)	1.01 (0.60-1.69)
Hotel Receptionists	4224	985	<5	0.79 (0.20-3.15)	0.38 (0.05-2.72)
Missing ISCO-08 code	--	59,574	306	1.26 (1.09-1.47)	0.96 (0.82-1.11)
Reference (all occupations with unlikely occupational SARSCoV-2 exposure)^a^		102,168	395	1.00	1.00

^a^Likelihood of occupational SARS-CoV-2 exposure according to a population-based international expert-rated job exposure matrix that assesses four measures of the number of close indoor contacts at work, two mitigation measures and two job insecurity measures, each rated on a scale from low (0) to high (3).

^b^Adjusted for sex and age (10-year groups).

^c^Adjusted for sex, age (10-year groups), education (3 groups), country of origin (4 categories), number of household members (0, 1, 2, 3, 4+), and COVID-19 vaccination (from date of second vaccination until end of follow-up).

^d^ISCO-08 codes 2221, 2211, 2212, 2213, 2219, 3251, 5321, 2264, 3212, 2634, 2269, 2261, 3211, 2222, 5322, 2635.

^e^ISCO-08 codes 5311, 2342, 2341, 2330, 2310, 2320.

^f^ISCO-08 codes 8332, 8331, 8321, 8311.

^g^ISCO-08 codes 5223, 5230, 1420, 3213, 7511.

^h^ISCO-08 codes 5120, 5131, 5132, 5411–5419, 9412, 9111, 5152, 5141, 5142 9411, 6113, 4224.

^i^Occupations are mutually exclusive.

In the educational sector, no elevated risk was observed overall (IRR 1.03; 95% CI: 0.86–1.23). However, among secondary education teachers some indication of elevated risk was noted IRR 1.31; 95% CI: 0.85–2.04).

For the transportation sector, an overall excess risk was observed (IRR 1.34; 95% CI: 1.10–1.65), with bus and tram drivers having the highest risk, IRR 1.68 (95% CI: 1.27–2.23). Car, taxi, and van drivers also appeared to have higher risks. Although the point estimate was elevated, the wide confidence intervals reflect limited statistical power due to a low number of hospital admissions.

No elevated risk was observed in the retail sales sector overall or in specific occupations. Similarly, for accommodation, food, building, personal and protective services, and recreational activity sectors, no elevated risks were noted overall or across specific occupations. However, there was a higher risk of COVID-19 admission among fast food preparers with an IRR of 1.76 (95% CI: 0.90–3.42) (Table 2).

Across an additional 62 professions with >500 employees, few occupations were associated with risk of COVID-19-related admission (S3 Table). We did observe excess risk among human resource managers (IRR 1.82; 95% CI: 0.97–3.42), while lower risk was noted among building electricians (IRR 0.52; 95% CI: 0.27–1.00), building frame and related trades workers (e.g., scaffolders, assemblers of prefabricated buildings/houses) (IRR 0.50; 95% CI: 0.25–1.00), as well as software developers (IRR 0.60; 95% CI: 0.39–0.93) (S3 Table).

Overall, when restricting analyses to participants with a diagnosis of COVID-19 in diagnostic position one (n = 2,124), IRRs were not markedly different (S4 Table), with the exception of primary school teachers (IRR 1.27; 95% CI: 0.77–2.11 versus IRR 1.03; 95% CI: 0.78–1.37), waiters and bartenders (IRR 1.42; 95% CI: 0.73–2.76 versus IRR 1.14; 95% CI: 0.61–2.14), and kitchen helpers (IRR 1.21; 95% CI: 0.83–1.75 versus IRR 1.07; 95% CI 0.76–1.50) where IRRs were somewhat higher. Conversely, occupations within the transportation sector were generally lower. In additional sensitivity analyses, only small changes were observed when using a case definition with higher specificity for COVID-19 (e.g., only using the ICD10 code U07.1, n = 2,443) (S5 Table).

## Discussion

In this Swedish register-based study we observed increased risks of COVID-19-related admission in many patient-facing occupations across the healthcare sector as well as in multiple occupations within the transportation sector, specifically bus and taxi drivers. However, no apparent excess risks were observed in the educational sector or retail sales sectors and a decreased risk was observed among electricians, some construction workers, and software developers. Despite the absence of widespread lockdowns in Sweden, the magnitude of occupational risks for childcare, education, retail, and service-oriented professions appeared broadly similar to those reported in neighboring countries with extensive lockdowns. However, such comparisons should be interpreted cautiously given differences in study design, background incidence, and other contextual factors [3,8,17].

Frequency and proximity of contact with infected persons remain key contributing factors to being infected with SARS-CoV-2 at the workplace. Our findings are in general agreement with earlier studies which demonstrated elevated risk of COVID-19 across multiple occupations in the healthcare and transportation sectors [3,5,6,8–10,18–23]. For example, a UK study in 900,000 National Health Service workers also reported elevated risks among nurses, care assistants, and health professionals, but also in dentists and midwifery professionals, a finding not observed in the present study [6]. Similarly, a Norwegian case-control study of around 3.5 million persons reported higher risks among nurses, physicians, dentists and physiotherapists [8]. A recent Danish register-based study of 2.4 million Danish employees, conducted by the present research group using comparable methodology, also found elevated risks for several occupations within healthcare, but also medical laboratory technicians, hospital porters, and psychologists [3]. In the present study, point estimates were elevated in nine of the 13 healthcare occupations, although, within some occupations, analyses were hampered by the low number of cases. Studies have shown that over the pandemic period the occupational risks in healthcare workers declined, which is likely explained by increased access and adequate use of personal protective equipment (PPE), testing frequency, as well as early access to vaccination [4,17,24]. Although risk estimates were generally elevated across healthcare professionals, our results do not suggest that Sweden’s limited lockdown strategy substantially altered the occupational risk of COVID-19-related hospital admission in this group. Given that healthcare professionals were largely exempt from restrictions in many countries, such differences in occupational risk between Sweden and countries with stricter lockdowns may not be expected [3].

The transportation sector also appeared to be important to virus acquisition and this is also in line with prior studies [3,7,8,10]. We observed the highest risk in bus and tram drivers, which highlights that these professions would have likely benefited from increased training in infection control guidelines as well as PPE.

We found no apparent elevated risk in the education sector overall, which agrees with some, but not all studies [4,7,8]. A study from Norway reported higher risks in the education sector, but only for the second wave of the pandemic (i.e., 18 July to 18 December 2020) [8]. Furthermore, a Swedish case control study with recruitment from October 2020 to December 2021, reported higher risk of both severe COVID-19 (based on hospital admissions) and SARS-CoV-2 infection (based on positive polymerase chain reaction test) among primary school teachers and early childhood educators compared to occupations with low risk of exposure [7]. Our findings of no overall elevated risk are in agreement with one Danish study, which also found no increased risk of hospital admissions for COVID-19 in the educational, retail sales sectors, and some service occupations [3]. However, the Danish study did observe elevated risks among childcare workers providing daytime care for 4–7-year-old children, whereas we found no elevated risk. Although secondary schools, universities, and some businesses implemented teleworking, Sweden generally remained open, which contrasts with other countries where many childcare centers as well as primary schools were closed or where teaching took place digitally from home. Our findings support the argument that perhaps primary school closures should be carefully considered and possibly avoided in certain contexts, particularly, due to the negative impacts on children’s mental health due to closures [25]. Nonetheless, evidence from other countries is mixed, and our study does not address infection risk among children. Decisions regarding primary school closures should be based on multiple considerations, including both potential impacts on transmission and the well-being of children.

Some occupations were associated with lower risk of COVID-19-related hospital admission compared to those with the lowest JEM sum score, namely electricians, some types of construction workers, and software developers. The lower risk among certain construction workers (e.g., building frame and trade workers) is in line with previous research and is likely related to outdoor working environments [7,26]. However, not all studies have shown this, for example a British and a US study both found that outdoor work was associated with elevated risk for SARS-CoV-2 infection [17,27]. For software developers, the option of working from home was encouraged by both employers and Swedish healthcare authorities, which likely resulted in fewer contacts with infected persons and consequently reduced the risk of infection. Lower risk of SARS-CoV-2 infection in many highly skilled occupations has also been reported by others, which likely had to do with early implementation of teleworking [3]. For example, in Skåne from quarter four 2020 to quarter two 2021 37–41% of those ages 17–74 years worked from home at least one day a week [28].

Our study has multiple strengths. Firstly, it constitutes a major extension of prior publications, as Sweden, being one of few countries with no sweeping mandatory lockdowns (particularly for childcare and primary school education), serves as an important benchmark for comparison. Secondly, we had access to regional and national registries with high accuracy, coverage, and validity pertaining to occupation as well as COVID-19 diagnoses. Thirdly, we were able to control for vaccination status.

Some weaknesses should be acknowledged. We likely missed some occupations that could be at risk due to the limited number of people (e.g., occupations with <500 employees). In addition, since we only accounted for occupation at a single time point (November 2019) one could expect some degree of misclassification since some employees would have started, stopped, or changed jobs during follow-up. Although we used four-digit level occupational codes, these potentially comprise multiple occupations leading to some truncation of occupations. Residual confounding from factors such as pre-existing chronic diseases, social contacts outside the household and workplace, or use of public transportation are additional sources of potential bias. Moreover, outside of healthcare professionals, it is unclear which occupations and individuals had access to and utilized PPE and could be a source of interaction. Since we use hospital-based admissions, we had no data on rapid antigen tests taken at home or other testing centers, and cases thus likely only constitutes more severe COVID-19. Moreover, these two outcomes are not interchangeable and equating them would be misleading. Hospital admissions reflect a more severe spectrum of COVID-19 and are influenced by factors beyond infection risk itself, including age and comorbidities. Consequently, our findings should not be interpreted as direct estimates of occupational risk of contracting COVID-19, but rather as estimates of occupational differences in the risk of progressing to a hospital admission with COVID-19. The intended causal pathway is that occupation influences infection risk, which in turn influences hospitalization; however, alternative pathways are also possible, such as occupation influencing healthcare-seeking behavior, which could independently affect hospitalization risk. Clarifying this distinction is important to avoid misinterpreting hospitalization risk as a direct surrogate for infection risk. We also had no way of disentangling infections acquired at work from those outside work, and exposure outside work or comorbidities are relevant for admission [23,29,30]. However, we adjusted for family size which likely limited this source of potential confounding. Also, multiple comparisons could result in spurious findings and some results should be interpreted with caution, particularly those with a limited number of cases (e.g., psychological therapists, dentists, and midwifery professionals). While we did not stratify by pandemic wave, variant, or vaccination period, our focus was on overall occupational differences across the full two-year period; future research could explore how temporal changes may have influenced these patterns. Lastly, the COVID-19-JEM utilized in the present study has not been validated, and was constructed using subjective expert ratings, and could result in exposure misclassification. Moreover, the JEM was developed shortly after the pandemic began, when knowledge about SARS-CoV-2 transmission and variants was uncomprehensive [18,31]. Although it effectively assesses occupational risk, it should be updated with current knowledge. For example, ventilation is now recognized as a crucial factor due to the major role of airborne transmission [32,33]. While considering work location (indoors vs. outdoors) is valuable, incorporating ventilation aspects would also provide additional insights. Nevertheless, limited and highly variable data on ventilation rates in different workplaces make it difficult to provide reliable job-level assessments.

In general, understanding the risk of occupationally acquired COVID-19 is complex and multifaceted as risk factors often present simultaneously in vulnerable populations. The risks can span workplace characteristics (e.g., ventilation), preventive actions (e.g., COVID-19 testing, ability to maintain distance, or availability of PPE), as well as the social environment (e.g., mandated lockdowns). Moreover, an employee’s sociodemographic characteristics may also influence mode of commuting as well as domestic environment, and these factors often change over time. Overall, the progression of the pandemic and the related occupational risks must be interpreted within the specific context of each country, particularly in the present study, as Sweden had no mandatory lockdowns.

In this Swedish register-based study, our findings support and extend previous work demonstrating elevated risk of COVID-19-related hospital admission among patient-facing healthcare professionals. Our findings also suggest that occupations outside the healthcare sector, namely public transportation workers, may also be at increased risk of COVID-19-related hospital admission. Overall, Sweden’s minimal lockdown measures did not appear to result in higher occupational risks for workers in childcare, education, retail, and service sectors.

## Supporting information

S1 Table List of reference occupations. (DOCX)

S2 Table Distribution of baseline characteristics of the study population by COVID-19 JEM sum score^a^ (N = 607,179). (DOCX)

S3 Table Risk of COVID-19 related hospital admission for non-referent 4-digit ISCO-08 occupations with >500 employees. Incidence rate ratios (IRR) with 95% confidence limits relative to employees in all occupations with unlikely occupational exposure to SARSCoV-2. (DOCX)

S4 Table Risk of COVID-19 related hospital admission, *based on diagnostic position one,* in relation to occupation. Incidence rate ratios (IRR) with 95% confidence limits (CI) relative to employees in all occupations with unlikely occupational exposure to SARS-CoV-2. (DOCX)

S5 Table Risk of ICD-10 code U07.1 only (*virus identified)* COVID-19 related hospital admission in relation to occupation. Incidence rate ratios (IRR) with 95% confidence limits (CI) relative to employees in all occupations with unlikely occupational exposure to SARS-CoV-2. (DOCX)
