# Supplementary material for: Occupational risk of COVID-19 related hospital admission in Skåne, Sweden: A register-based cohort study
Source: PLoS One. 2025 Nov 4;20(11):e0335662. doi: 10.1371/journal.pone.0335662 (PMC12585036; doi:10.1371/journal.pone.0335662)
Supplement: S1 Table — (DOCX) [file pone.0335662.s001.docx]

| Table S1. List of reference occupations. |  |  |
| --- | --- | --- |
| Occupation | **ISCO-08 code** | **Frequency** |
| Production clerks | 4322 | 12566 |
| Commercial sales representatives | 3322 | 11337 |
| Statistical, mathematical and related associate professionals | 3314 | 10049 |
| Policy administration professionals | 2422 | 9258 |
| Policy and planning managers | 1213 | 7060 |
| Advertising and marketing professionals | 2431 | 4878 |
| Accounting and bookkeeping clerks | 4311 | 4773 |
| Accounting associate professionals | 3313 | 3237 |
| Legal and Related Associate Professionals | 3411 | 2850 |
| Credit and loans officers | 3312 | 2573 |
| Personnel and careers professionals | 2423 | 2548 |
| Trade brokers | 3324 | 2341 |
| Education methods specialists | 2351 | 2221 |
| Systems analysts | 2511 | 2126 |
| Public relations professionals | 2432 | 2064 |
| Procurement and buyers | 3323 | 2050 |
| Sales and marketing managers | 1221 | 2043 |
| Payroll clerks | 4313 | 1731 |
| Accountants | 2411 | 1628 |
| Transport clerks | 4323 | 1512 |
| Clerical support workers not elsewhere classified | 4419 | 1473 |
| Government social benefits officials | 3353 | 1239 |
| Employment agents and contractors | 3333 | 1129 |
| Insurance representatives | 3321 | 1123 |
| Stock clerks | 4321 | 1031 |
| Building architects | 2161 | 992 |
| General office clerks | 4110 | 873 |
| Research and development managers | 1223 | 849 |
| Finance managers | 1211 | 826 |
| Advertising and public relations managers | 1222 | 799 |
| Government tax and excise officials | 3352 | 640 |
| Library clerks | 4411 | 480 |
| Financial analysts | 2413 | 427 |
| Telephone switchboard operators | 4223 | 390 |
| Statistical, finance and insurance clerks | 4312 | 356 |
| Conference and event planners | 3332 | 305 |
| Landscape architects | 2162 | 176 |
| Town and traffic planners | 2164 | 91 |
| Economists | 2631 | 76 |
| Securities and finance dealers and brokers | 3311 | 48 |
