# Supplementary material for: Occupational risk of COVID-19 related hospital admission in Skåne, Sweden: A register-based cohort study
Source: PLoS One. 2025 Nov 4;20(11):e0335662. doi: 10.1371/journal.pone.0335662 (PMC12585036; doi:10.1371/journal.pone.0335662)
Supplement: S2 Table — (DOCX) [file pone.0335662.s002.docx]

| Table S2. Distribution of baseline characteristics of the study population by COVID-19 JEM sum score^a^ (N=607,179).^b^ | | | | |
| --- | --- | --- | --- | --- |
| Characteristic | **Reference**  **(JEM sum score = 0)**  **n = 102,168** | **At risk occupations**  **(JEM sum score 1-24)**  **n = 445,437** | **Missing**  **occupational codes**  **n = 59,574** |  |
| Sex | | | | |
| Males | 46.8 | 51.0 | 59.4 |  |
| Females | 53.2 | 49.0 | 40.6 |  |
| Age (years) |  |  |  |  |
| 20 - <30 | 14.1 | 19.2 | 23.6 |  |
| 30 - <40 | 23.3 | 23.3 | 23.1 |  |
| 40 - <50 | 26.9 | 23.4 | 19.9 |  |
| 50 - <60 | 25.0 | 22.5 | 18.5 |  |
| ≥60 | 10.7 | 11.6 | 14.9 |  |
| Education | | | | |
| Short | 5.4 | 8.9 | 15.0 |  |
| Medium | 47.1 | 52.4 | 53.7 |  |
| Long | 47.5 | 38.7 | 31.3 |  |
| Country of birth | | | | |
| Sweden | 85.5 | 77.4 | 66.3 |  |
| Other western countries | 5.2 | 7.2 | 10.8 |  |
| Eastern Europe | 4.0 | 5.9 | 6.4 |  |
| Other | 5.3 | 9.5 | 16.5 |  |
| Number of household members | | | | |
| 1 | 25.2 | 29.2 | 34.2 |  |
| 2 | 27.5 | 27.3 | 26.6 |  |
| 3 | 18.5 | 17.7 | 16.6 |  |
| ≥4 | 28.8 | 25.8 | 22.6 |  |
| Second Covid-19 vaccination obtained | | | | |
| 1.01.2021 – 30.06.2021 | 22.3 | 29.3 | 21.5 |  |
| 1.07.2021 – 14.12.2021 | 64.4 | 53.1 | 52.4 |  |
| ^a^ Likelihood of occupational SARS-CoV-2 exposure according to a population-based international expert-rated job exposure matrix that assesses four measures of number of close indoor contacts at work, two mitigation measures and two job insecurity measures, each rated on a scale from low (0) to high (3).  ^b^ Column percentages unless otherwise stated. | | | | |
