# Supplementary material for: Occupational risk of COVID-19 related hospital admission in Skåne, Sweden: A register-based cohort study
Source: PLoS One. 2025 Nov 4;20(11):e0335662. doi: 10.1371/journal.pone.0335662 (PMC12585036; doi:10.1371/journal.pone.0335662)
Supplement: S3 Table — (DOCX) [file pone.0335662.s003.docx]

| Table S3. Risk of COVID-19 related hospital admission for non-referent 4-digit ISCO-08 occupations with >500 employees. Incidence rate ratios (IRR) with 95% confidence limits relative to employees in all occupations with unlikely occupational exposure to SARSCoV-2^a^. | | | | | |
| --- | --- | --- | --- | --- | --- |
| Occupation | **ISCO-08 code** | **Employees**  **(n)** | **COVID-19 admissions**  **(n)** | **Crude**  **adjustment**  **IRR (95% CI)^b^** | **Fully adjusted**  **IRR (95% CI)^c^** |
| Software Developers | 2512 | 9,519 | 21 | 0.55 (0.35-0.85) | 0.60 (0.39-0.93) |
| Management and Organization Analysts | 2421 | 4,165 | 9 | 0.52 (0.27-1.01) | 0.58 (0.30-1.13) |
| Motor Vehicle Mechanics and Repairers | 7231 | 4,135 | 24 | 1.26 (0.84-1.91) | 1.03 (0.68-1.56) |
| Building and Related Electricians | 7411 | 4,095 | 9 | 0.52 (0.27-1.00) | 0.52 (0.27-1.00) |
| Contact Centre Information Clerks | 4222 | 3,608 | 9 | 0.87 (0.45-1.68) | 0.85 (0.44-1.64) |
| Metal Working Machine Tool Setters and Operators | 7223 | 3,443 | 23 | 1.40 (0.92-2.14) | 1.06 (0.69-1.62) |
| Financial and Investment Advisers | 2412 | 3,182 | 9 | 0.73 (0.38-1.42) | 0.80 (0.41-1.55) |
| Building Frame and Related Trades Workers Not Elsewhere Classified | 7119 | 3,143 | 8 | 0.59 (0.29-1.20) | 0.50 (0.25-1.00) |
| Civil Engineering Technicians | 3112 | 2,998 | 6 | 0.45 (0.20-1.02) | 0.48 (0.22-1.08) |
| Food and Related Products Machine Operators | 8160 | 2,986 | 23 | 2.00 (1.31-3.05) | 1.27 (0.83-1.93) |
| Physical and Engineering Science  Technicians | 3119 | 2,711 | 10 | 0.84 (0.45-1.57) | 0.86 (0.46-1.60) |
| Stationary Plant and Machine Operators Not Elsewhere Classified | 8189 | 2,610 | 16 | 1.40 (0.85-2.32) | 1.07 (0.65-1.77) |
| Manufacturing Managers | 1321 | 2,454 | 7 | 0.55 (0.26-1.16) | 0.56 (0.27-1.19) |
| Waiters | 5131 | 2,386 | 6 | 1.02 (0.45-2.28) | 0.84 (0.37-1.89) |
| Prison Guards | 5413 | 2,294 | 10 | 1.22 (0.65-2.28) | 1.06 (0.57-1.99) |
| Managing Directors and Chief Executives | 1120 | 2,270 | 12 | 0.92 (0.52-1.63) | 0.98 (0.55-1.74) |
| Engineering Professionals Not Elsewhere Classified | 2149 | 2,248 | 8 | 0.81 (0.40-1.62) | 0.88 (0.44-1.78) |
| Electronics Engineering Technicians | 3114 | 2,188 | 10 | 0.94 (0.50-1.76) | 0.99 (0.53-1.85) |
| Chemical and Physical Science Technicians | 3111 | 2,119 | 7 | 0.82 (0.39-1.73) | 0.69 (0.31-1.56) |
| Mechanical Engineers | 2144 | 2,081 | 8 | 0.90 (0.45-1.81) | 1.00 (0.49-2.01) |
| Mail Carriers and Sorting Clerks | 4412 | 2,065 | <5 | 0.41 (0.13-1.27) | 0.39 (0.13-1.23) |
| Civil Engineers | 2142 | 2,064 | <5 | 0.47 (0.18-1.27) | 0.53 (0.20-1.41) |
| Hairdressers | 5141 | 2,048 | 10 | 1.57 (0.84-2.95) | 1.06 (0.57-2.00) |
| Information and Communications  Technology User Support | 3512 | 1,991 | <5 | 0.52 (0.20-1.40) | 0.56 (0.21-1.49) |
| Painters and Related Workers | 7131 | 1,942 | 11 | 1.15 (0.63-2.10) | 0.99 (0.54-1.80) |
| Dental assistants and Therapists | 3251 | 1,880 | 10 | 1.67 (0.89-3.13) | 1.45 (0.77-2.73) |
| Legal Professionals Not Elsewhere  Classified | 2619 | 1,866 | 7 | 1.10 (0.52-2.33) | 1.33 (0.63-2.80) |
| Software and Applications Developers and Analysts Not Elsewhere | 2519 | 1,751 | <5 | 0.55 (0.21-1.48) | 0.55 (0.21-1.49) |
| Hand Packers | 9321 | 1,632 | 12 | 2.06 (1.16-3.65) | 1.41 (0.79-2.51) |
| Real Estate Agents and Property Managers | 3334 | 1,573 | <5 | 0.62 (0.23-1.67) | 0.67 (0.25-1.78) |
| Psychologists | 2634 | 1,450 | <5 | 0.19 (0.03-1.34) | 0.22 (0.03-1.54) |
| Bricklayers and Related Workers | 7112 | 1,421 | <5 | 0.63 (0.23-1.68) | 0.51 (0.19-1.37) |
| Welders and Flame Cutters | 7212 | 1,415 | 8 | 1.13 (0.56-2.28) | 0.91 (0.45-1.84) |
| Retail and Wholesale Trade Managers | 1420 | 1,402 | 7 | 1.01 (0.48-2.14) | 0.94 (0.44-1.98) |
| Human Resource Managers | 1212 | 1,304 | 10 | 1.70 (0.91-3.18) | 1.82 (0.97-3.42) |
| Plastic Products Machine Operators | 8142 | 1,227 | <5 | 0.57 (0.18-1.78) | 0.40 (0.13-1.25) |
| Child Care Services Managers | 1341 | 1,196 | 6 | 1.21 (0.54-2.70) | 1.33 (0.59-2.98) |
| Assemblers Not Elsewhere Classified | 8219 | 1,180 | 7 | 1.54 (0.73-3.26) | 1.19 (0.56-2.52) |
| Clearing and Forwarding Agents | 3331 | 1,178 | <5 | 0.68 (0.22-2.11) | 0.69 (0.22-2.16) |
| Journalists | 2642 | 1,062 | <5 | 0.22 (0.03-1.59) | 0.27 (0.04-1.92) |
| Electrical and Electronic Equipment  Assemblers | 8212 | 1,055 | 7 | 1.46 (0.69-3.08) | 1.07 (0.51-2.27) |
| Police Officers | 5412 | 1,044 | 5 | 1.26 (0.52-3.05) | 1.17 (0.49-2.84) |
| Messengers, Package Deliverers and  Luggage Porters | 9621 | 1,028 | <5 | 0.44 (0.11-1.78) | 0.31 (0.08-1.25) |
| Administrative and Executive Secretaries | 3343 | 1,017 | <5 | 1.11 (0.41-2.97) | 1.08 (0.40-2.89) |
| Musicians, Singers and Composers | 2652 | 943 | <5 | 0.46 (0.12-1.85) | 0.52 (0.13-2.08) |
| Civil Engineering Laborers | 9312 | 941 | <5 | 0.79 (0.25-2.45) | 0.61 (0.20-1.90) |
| Industrial and Production Engineers | 2141 | 878 | <5 | 1.17 (0.44-3.12) | 0.92 (0.30-2.88) |
| Lifting Truck Operators | 8344 | 857 | <5 | 0.50 (0.12-2.01) | 0.41 (0.10-1.67) |
| Special Needs Teachers | 2352 | 767 | <5 | 0.37 (0.05-2.64) | 0.42 (0.06-2.97) |
| Metal Processing Plant Operators | 8121 | 698 | <5 | 1.20 (0.45-3.20) | 0.82 (0.31-2.20) |
| Lawyers | 2611 | 694 | <5 | 0.64 (0.16-2.55) | 0.82 (0.20-3.29) |
| Mechanical Machinery Assemblers | 8211 | 691 | <5 | 0.97 (0.31-3.03) | 0.67 (0.21-2.09) |
| Medical Imaging and Equipment Operators | 3211 | 685 | 5 | 1.81 (0.75-4.37) | 1.79 (0.74-4.34) |
| Social Work Associate Professionals | 3412 | 680 | <5 | 1.60 (0.51-4.99) | 1.68 (0.54-5.23) |
| Chemical Engineers | 2145 | 665 | <5 | 1.12 (0.36-3.47) | 1.24 (0.40-3.85) |
| Armed Forces Occupations, Other Ranks | 0310 | 650 | 0 | -- | -- |
| Electrical Mechanics and Fitters | 7412 | 648 | 5 | 1.53 (0.63-3.71) | 1.49 (0.62-3.61) |
| Personal Care Workers in Health  Services | 5329 | 611 | <5 | 0.54 (0.08-3.85) | 0.42 (0.06-2.99) |
| Plumbers and Pipe Fitters | 7126 | 597 | 0 | -- | -- |
| Mechanical Engineering Technicians | 3115 | 572 | 0 | -- | -- |
| Chemical Products Plant and Machine Operators | 8131 | 523 | <5 | 1.03 (0.26-4.12) | 0.78 (0.20-3.15) |
| Occupations with less than 500 employees |  | 26,756 | 112 | 0.99 (0.80-1.22) | 0.92 (0.74-1.13) |
| Reference (all occupations with unlikely occupational SARSCoV-2 exposure)^a^ |  | 102,168 | 395 | 1.00 | 1.00 |
| ^a^ Likelihood of occupational SARS-CoV-2 exposure according to a population-based international expert-rated job exposure matrix that assesses four measures of the number of close indoor contacts at work, two mitigation measures and two job insecurity measures, each rated on a scale from low (0) to high (3).  ^b^ Adjusted for sex and age (10-year groups).  ^c^ Adjusted for sex, age (10-year groups), education (3 groups), country of origin (4 categories), number of household members (0, 1, 2, 3, 4+), and COVID-19 vaccination (from date of second vaccination until end of follow-up). | | | | | |
