# Supplementary material for: Occupational risk of COVID-19 related hospital admission in Skåne, Sweden: A register-based cohort study
Source: PLoS One. 2025 Nov 4;20(11):e0335662. doi: 10.1371/journal.pone.0335662 (PMC12585036; doi:10.1371/journal.pone.0335662)
Supplement: S4 Table — (DOCX) [file pone.0335662.s004.docx]

| Table S4. Risk of COVID-19 related hospital admission, *based on diagnostic position one,* in relation to occupation. Incidence rate ratios (IRR) with 95% confidence limits (CI) relative to employees in all occupations with unlikely occupational exposure to SARS-CoV-2^a^. | | | | | |
| --- | --- | --- | --- | --- | --- |
| Occupation | **ISCO-08 code** | **Employees**  **(n)** | **COVID-19 admissions**  **(n)** | **Crude**  **adjustment**  **IRR (95% CI)^b^** | **Fully adjusted**  **IRR (95% CI)^c^** |
| HEALTHCARE | | | | | |
| Healthcare overall | ^d^ | 77,022 | 305 | 1.67 (1.42-1.96) | 1.38 (1.17-1.63) |
| Nursing Professionals | 2221 | 13,225 | 46 | 1.47 (1.07-2.01) | 1.71 (1.24-2.35) |
| Medical Practitioners | 2211, 2212, 2213, 2219 | 6,236 | 22 | 1.11 (0.72-1.71) | 1.05 (0.68-1.63) |
| Healthcare Assistants | 5321 | 42,596 | 170 | 1.77 (1.46-2.15) | 1.34 (1.10-1.63) |
| Physiotherapists | 2264 | 1,717 | 6 | 1.42 (0.63-3.18) | 1.72 (0.77-3.88) |
| Medical Laboratory Technicians | 3212 | 1,528 | 5 | 1.38 (0.57-3.34) | 1.22 (0.50-2.95) |
| Psychological Therapists | 2634 | 1,450 | <5 | 0.25 (0.04-1.78) | 0.29 (0.04-2.07) |
| Recreational Therapists | 2269 | 1,452 | 6 | 1.81 (0.81-4.07) | 2.07 (0.92-4.66) |
| Dentist | 2261 | 835 | <5 | 0.80 (0.20-3.23) | 0.70 (0.17-2.81) |
| Dental Assistants and Therapists | 3251 | 1,880 | 7 | 1.68 (0.79-3.57) | 1.46 (0.69-3.10) |
| X-ray Technicians | 3211 | 685 | <5 | 1.84 (0.69-4.94) | 1.83 (0.68-4.92) |
| Midwifery Professionals | 2222 | 765 | <5 | 0.55 (0.08-3.95) | 0.64 (0.09-4.59) |
| Nursing Aides (private homes) | 5322 | 8,281 | 48 | 2.29 (1.69-3.10) | 1.47 (1.07-2.00) |
| Other Hospital related activities | 2635 | 2,608 | 9 | 1.32 (0.68-2.56) | 1.27 (0.65-2.46) |
| EDUCATION | | | | | |
| Overall Education | ^e^ | 49,111 | 135 | 1.09 (0.89-1.34) | 1.03 (0.84-1.27) |
| Childcare Workers | 5311 | 11,678 | 37 | 1.69 (1.19-2.38) | 1.11 (0.78-1.57) |
| Preschool Teachers | 2342 | 11,420 | 24 | 0.95 (0.63-1.44) | 1.06 (0.69-1.61) |
| Primary School Teachers | 2341 | 15,163 | 48 | 1.23 (0.91-1.67) | 1.16 (0.86-1.58) |
| Secondary Education Teachers | 2330 | 4,146 | 16 | 1.18 (0.71-1.94) | 1.27 (0.77-2.11) |
| University and Higher Education Teachers | 2310 | 5,438 | 5 | 0.28 (0.12-0.68) | 0.32 (0.13-0.77) |
| Vocational Education Teachers | 2320 | 1,266 | 5 | 0.97 (0.40-2.34) | 1.05 (0.43-2.54) |
| TRANSPORT | | | | | |
| Overall Transport | ^f^ | 13,070 | 105 | 1.83 (1.46-2.29) | 1.21 (0.96-1.52) |
| Heavy Truck and Lorry Drivers | 8332 | 7,158 | 31 | 1.02 (0.70-1.47) | 0.90 (0.62-1.30) |
| Bus and Tram Drivers | 8331 | 3,069 | 53 | 3.45 (2.57-4.62) | 1.64 (1.22-2.21) |
| Car, Taxi and Van Drivers | 8321 | 2,249 | 19 | 1.96 (1.23-3.12) | 1.07 (0.67-1.71) |
| Locomotive Engine Drivers | 8311 | 594 | <5 | 0.80 (0.20-3.21) | 0.89 (0.22-3.59) |
| RETAIL SALES | | | | | |
| Overall Retail Sales | ^g^ | 17,708 | 47 | 1.12 (0.83-1.53) | 1.00 (0.73-1.36) |
| Shop Sales Assistants | 5223 | 13,418 | 35 | 1.22 (0.86-1.73) | 1.15 (0.81-1.64) |
| Cashiers and Ticket Clerks | 5230 | 1,547 | <5 | 0.35 (0.05-2.49) | 0.26 (0.04-1.89) |
| Retail Trade Managers | 1420 | 1,402 | 6 | 1.04 (0.46-2.34) | 0.94 (0.42-2.10) |
| Pharmaceutical Technicians and Assistants | 3213 | 509 | 0 | -- | -- |
| Butchers and Fishmongers | 7511 | 832 | 5 | 1.46 (0.60-3.54) | 0.95 (0.39-2.30) |
| ACCOMODATION, FOOD, BUILDING, PERSONAL AND  PROTECTIVE SERVICES, RECREATION ACTIVITY | | | | | |
| Overall Accommodation, Food, Building, Personal and Protective Services, Recreation Activity | ^h^ | 27,986 | 135 | 1.68 (1.37-2.06) | 0.95 (0.77-1.18) |
| Cooks | 5120 | 5,087 | 21 | 1.51 (0.97-2.34) | 0.94 (0.59-1.50) |
| Waiters and Bartenders | 5131, 5132 | 3,135 | 8 | 1.70 (0.88-3.30) | 1.42 (0.73-2.76) |
| Protective Service Workers | 5411-5419 | 4,465 | 15 | 0.82 (0.45-1.50) | 0.75 (0.41-1.37) |
| Kitchen Helpers | 9412 | 6,967 | 33 | 2.27 (1.58-3.25) | 1.21 (0.83-1.75) |
| Cleaners and Helpers | 9111 | 10,749 | 52 | 1.89 (1.40-2.54) | 0.86 (0.63-1.16) |
| Building Caretakers | 5152 | 5,183 | 29 | 1.21 (0.82-1.77) | 0.94 (0.64-1.38) |
| Hairdressers and Cosmetologists | 5141,5142 | 2,990 | 11 | 1.72 (0.94-3.14) | 1.11 (0.61-2.03) |
| Fast Food Preparers | 9411 | 544 | 9 | 4.20 (1.98-8.89) | 1.58 (0.74-3.35) |
| Gardeners and Horticultural Growers | 6113 | 2,644 | 11 | 1.04 (0.57-1.89) | 0.87 (0.48-1.59) |
| Hotel Receptionists | 4224 | 985 | 0 | -- | -- |
| Missing ISCO-08 code | -- | 59,574 | 249 | 1.27 (1.08-1.50) | 0.95 (0.80-1.13) |
| Reference (all occupations with unlikely occupational SARSCoV-2 exposure)^a^ |  | 102,168 | 308 | 1.00 | 1.00 |
| ^a^ Likelihood of occupational SARS-CoV-2 exposure according to a population-based international expert-rated job exposure matrix that assesses four measures of the number of close indoor contacts at work, two mitigation measures and two job insecurity measures, each rated on a scale from low (0) to high (3).  ^b^ Adjusted for sex and age (10-year groups).  ^c^ Adjusted for sex, age (10-year groups), education (3 groups), country of origin (4 categories), number of household members (0, 1, 2, 3, 4+), and COVID-19 vaccination (from date of second vaccination until end of follow-up).  ^d^ ISCO-08 codes 2221, 2211, 2212, 2213, 2219, 3251, 5321, 2264, 3212, 2634, 2269, 2261, 3211, 2222, 5322, 2635.  ^e^ ISCO-08 codes 5311, 2342, 2341, 2330, 2310, 2320.  ^f^ ISCO-08 codes 8332, 8331, 8321, 8311.  ^g^ ISCO-08 codes 5223, 5230, 1420, 3213, 7511.  ^h^ ISCO-08 codes 5120, 5131, 5132, 5411-5419, 9412, 9111, 5152, 5141, 5142 9411, 6113, 4224. | | | | | |
