# Supplementary material for: Occupational risk of COVID-19 related hospital admission in Skåne, Sweden: A register-based cohort study
Source: PLoS One. 2025 Nov 4;20(11):e0335662. doi: 10.1371/journal.pone.0335662 (PMC12585036; doi:10.1371/journal.pone.0335662)
Supplement: S5 Table — (DOCX) [file pone.0335662.s005.docx]

| Table S5. Risk of ICD-10 code U07.1 only (*virus identified)* COVID-19 related hospital admission in relation to occupation. Incidence rate ratios (IRR) with 95% confidence limits (CI) relative to employees in all occupations with unlikely occupational exposure to SARS-CoV-2^a^. | | | | | |
| --- | --- | --- | --- | --- | --- |
| Occupation | **ISCO-08 code** | **Employees**  **(n)** | **COVID-19 admissions**  **(n)** | **Crude**  **adjustment**  **IRR (95% CI)^b^** | **Fully adjusted**  **IRR (95% CI)^c^** |
| HEALTHCARE | | | | | |
| Healthcare overall | ^d^ | 77,022 | 363 | 1.64 (1.41-1.91) | 1.38 (1.18-1.60) |
| Nursing Professionals | 2221 | 13,225 | 57 | 1.50 (1.13-1.99) | 1.74 (1.30-2.31) |
| Medical Practitioners | 2211, 2212, 2213, 2219 | 6,236 | 27 | 1.18 (0.80-1.74) | 1.14 (0.77-1.69) |
| Healthcare Assistants | 5321 | 42,596 | 201 | 1.72 (1.44-2.06) | 1.31 (1.10-1.57) |
| Physiotherapists | 2264 | 1,717 | 7 | 1.42 (0.63-3.18) | 1.68 (0.79-3.56) |
| Medical Laboratory Technicians | 3212 | 1,528 | 7 | 1.60 (0.76-3.38) | 1.43 (0.67-3.02) |
| Psychological Therapists | 2634 | 1,450 | <5 | 0.21 (0.03-1.50) | 0.24 (0.03-1.74) |
| Recreational Therapists | 2269 | 1,452 | 5 | 1.24 (0.51-3.01) | 1.42 (0.59-3.45) |
| Dentist | 2261 | 835 | <5 | 0.69 (0.17-2.78) | 0.61 (0.15-2.44) |
| Dental Assistants and Therapists | 3251 | 1,880 | 9 | 1.76 (0.90-3.41) | 1.53 (0.79-2.97) |
| X-ray Technicians | 3211 | 685 | 5 | 1.99 (0.82-4.81) | 1.98 (0.82-4.78) |
| Midwifery Professionals | 2222 | 765 | <5 | 0.90 (0.22-3.63) | 1.04 (0.26-4.20) |
| Nursing Aides (private homes) | 5322 | 8,281 | 58 | 2.33 (1.76-3.07) | 1.53 (1.15-2.03) |
| Other Hospital related activities | 2635 | 2,608 | 9 | 1.10 (0.57-2.14) | 1.07 (0.55-2.08) |
| EDUCATION | | | | | |
| Overall Education | ^e^ | 49,111 | 160 | 1.08 (0.90-1.31) | 1.03 (0.85-1.24) |
| Childcare Workers | 5311 | 11,678 | 44 | 1.61 (1.17-2.21) | 1.08 (0.79-1.49) |
| Preschool Teachers | 2342 | 11,420 | 33 | 1.07 (0.74-1.53) | 1.18 (0.82-1.70) |
| Primary School Teachers | 2341 | 15,163 | 53 | 1.14 (0.86-1.53) | 1.09 (0.82-1.47) |
| Secondary Education Teachers | 2330 | 4,146 | 17 | 1.08 (0.66-1.76) | 1.17 (0.72-1.91) |
| University and Higher Education Teachers | 2310 | 5,438 | 8 | 0.39 (0.19-0.79) | 0.44 (0.22-0.89) |
| Vocational Education Teachers | 2320 | 1,266 | 5 | 0.86 (0.35-2.07) | 0.93 (0.38-2.24) |
| TRANSPORT | | | | | |
| Overall Transport | ^f^ | 13,070 | 126 | 1.98 (1.61-2.43) | 1.33 (1.08-1.64) |
| Heavy Truck and Lorry Drivers | 8332 | 7,158 | 41 | 1.21 (0.87-1.67) | 1.06 (0.77-1.47) |
| Bus and Tram Drivers | 8331 | 3,069 | 59 | 3.49 (2.64-4.60) | 1.70 (1.28-2.25) |
| Car, Taxi and Van Drivers | 8321 | 2,249 | 23 | 2.14 (1.40-3.26) | 1.20 (0.78-1.84) |
| Locomotive Engine Drivers | 8311 | 594 | <5 | 1.07 (0.34-3.34) | 1.18 (0.38-3.69) |
| RETAIL SALES | | | | | |
| Overall Retail Sales | ^g^ | 17,708 | 56 | 1.13 (0.85-1.49) | 1.01 (0.76-1.34) |
| Shop Sales Assistants | 5223 | 13,418 | 42 | 1.22 (0.89-1.68) | 1.15 (0.83-1.60) |
| Cashiers and Ticket Clerks | 5230 | 1,547 | <5 | 0.28 (0.04-2.03) | 0.22 (0.03-1.57) |
| Retail Trade Managers | 1420 | 1,402 | 7 | 1.08 (0.51-2.28) | 0.98 (0.46-2.07) |
| Pharmaceutical Technicians and Assistants | 3213 | 509 | 0 | -- | -- |
| Butchers and Fishmongers | 7511 | 832 | 6 | 1.56 (0.70-3.50) | 1.03 (0.46-2.32) |
| ACCOMODATION, FOOD, BUILDING, PERSONAL AND  PROTECTIVE SERVICES, RECREATION ACTIVITY | | | | | |
| Overall Accommodation, Food, Building, Personal and Protective Services, Recreation Activity | ^h^ | 27,986 | 153 | 1.63 (1.35-1.97) | 0.93 (0.77-1.14) |
| Cooks | 5120 | 5,087 | 23 | 1.40 (0.92-2.13) | 0.86 (0.55-1.35) |
| Waiters and Bartenders | 5131, 5132 | 3,135 | 8 | 1.38 (0.71-2.67) | 1.17 (0.60-2.27) |
| Protective Service Workers | 5411-5419 | 4,465 | 16 | 1.03 (0.63-1.70) | 0.94 (0.57-1.55) |
| Kitchen Helpers | 9412 | 6,967 | 36 | 2.03 (1.44-2.87) | 1.12 (0.79-1.59) |
| Cleaners and Helpers | 9111 | 10,749 | 63 | 1.91 (1.46-2.50) | 0.89 (0.68-1.18) |
| Building Caretakers | 5152 | 5,183 | 31 | 1.16 (0.81-1.68) | 0.91 (0.63-1.32) |
| Hairdressers and Cosmetologists | 5141,5142 | 2,990 | 11 | 1.41 (0.77-2.57) | 0.94 (0.51-1.72) |
| Fast Food Preparers | 9411 | 544 | 8 | 4.16 (2.06-8.39) | 1.61 (0.80-3.27) |
| Gardeners and Horticultural Growers | 6113 | 2,644 | 13 | 1.09 (0.63-1.89) | 0.92 (0.53-1.60) |
| Hotel Receptionists | 4224 | 985 | 0 | -- | -- |
| Missing ISCO-08 code | -- | 59,574 | 282 | 1.26 (1.08-1.47) | 0.95 (0.80-1.13) |
| Reference (all occupations with unlikely occupational SARSCoV-2 exposure)^a^ |  | 102,168 | 359 | 1.00 | 1.00 |
| ^a^ Likelihood of occupational SARS-CoV-2 exposure according to a population-based international expert-rated job exposure matrix that assesses four measures of the number of close indoor contacts at work, two mitigation measures and two job insecurity measures, each rated on a scale from low (0) to high (3).  ^b^ Adjusted for sex and age (10-year groups).  ^c^ Adjusted for sex, age (10-year groups), education (3 groups), country of origin (4 categories), number of household members (0, 1, 2, 3, 4+), and COVID-19 vaccination (from date of second vaccination until end of follow-up).  ^d^ ISCO-08 codes 2221, 2211, 2212, 2213, 2219, 3251, 5321, 2264, 3212, 2634, 2269, 2261, 3211, 2222, 5322, 2635.  ^e^ ISCO-08 codes 5311, 2342, 2341, 2330, 2310, 2320.  ^f^ ISCO-08 codes 8332, 8331, 8321, 8311.  ^g^ ISCO-08 codes 5223, 5230, 1420, 3213, 7511.  ^h^ ISCO-08 codes 5120, 5131, 5132, 5411-5419, 9412, 9111, 5152, 5141, 5142 9411, 6113, 4224. | | | | | |
